# Taxonomic novelties and phylogenetic insights into Cainiaceae (Xylariales, Sordariomycetes) associated with Poaceae hosts from Southwestern China

**DOI:** 10.3897/mycokeys.128.178090

**Published:** 2026-02-02

**Authors:** Lakmali S. Dissanayake, Sajeewa S.N. Maharachchikumbura, Diana S. Marasinghe, Ying Gao, Turki KH. Faraj, Jianchu Xu, Dhanushka N. Wanasinghe

**Affiliations:** 1 CAS Key Laboratory for Plant Biodiversity and Biogeography of East Asia (KLPB), Kunming Institute of Botany, Chinese Academy of Science, Kunming 650201, Yunnan, China Centre for Mountain Futures (CMF), Kunming Institute of Botany, Chinese Academy of Sciences Kunming China; 2 Centre for Mountain Futures (CMF), Kunming Institute of Botany, Chinese Academy of Sciences, Kunming, 650201, China Kunming Institute of Botany, Chinese Academy of Science Kunming China; 3 School of Life Science and Technology, University of Electronic Science and Technology of China, Chengdu 611731, China King Saud University Riyadh Saudi Arabia; 4 Engineering Research Center of Southwest Bio-Pharmaceutical Resources, Ministry of Education, Guizhou University, Guiyang 550025, China Guizhou University Guiyang China; 5 Department of Soil Science, College of Food and Agriculture Sciences, King Saud University, P.O. Box 145111, Riyadh 11362, Saudi Arabia University of Electronic Science and Technology of China Chengdu China

**Keywords:** Ascomycota, grassland fungi, host specificity, morphological characterization, multigene phylogeny

## Abstract

Cainiaceae (Xylariales, Sordariomycetes) includes fungi primarily associated with hosts of the Poaceae and Arecaceae families. Recent studies have advanced the taxonomy of this family, highlighting its ecological and morphological diversity. Investigations focusing on microfungi associated with bamboo and other grass hosts in biodiversity-rich regions, such as Sichuan and Yunnan Provinces in China, have notably increased, driven by a growing recognition of their ecological and taxonomic importance. Fungal sampling was conducted on bamboo and related Poaceae hosts across various locations in Sichuan and Yunnan Provinces. Morphological characteristics were observed and recorded. Multilocus phylogenetic analyses, using the ITS and LSU regions, were conducted to determine phylogenetic relationships and confirm taxonomic placements. We introduce two new species and document one previously known species of *Arecophila*, and also describe two new species in *Amphibambusa* and *Longiappendispora*. Detailed morphological descriptions, illustrations and phylogenetic trees clearly delineate these taxa, highlighting their distinct affiliations with existing species. This study enriches the current understanding of fungal biodiversity within Cainiaceae, emphasizing their host associations and ecological specificity. Our findings underline the importance of continued fungal exploration in grassland ecosystems and contribute data towards stabilising the taxonomy of the Cainiaceae.

## Introduction

The family Cainiaceae was established by Krug (1978) within Xylariales (Sordariomycetes), designating *Cainia* as the type genus. The systematic position of the Cainiaceae has undergone several revisions. Hongsanan et al. (2017) treated the family as *incertae sedis* within Xylariomycetidae, whereas later studies (Hyde et al. 2020, 2024; Samarakoon et al. 2022) placed it in Xylariales. Phylogenomic evidence further reinforces its position in Xylariales (Chen et al. 2023). Using combined morpho-molecular approaches, Maharachchikumbura et al. (2015, 2016) accepted five genera in Cainiaceae, *viz*., *Amphibambusa*, *Arecophila*, *Atrotorquata*, *Cainia* and *Seynesia*. Mapook et al. (2020) introduced *Longiappendispora* to accommodate species with distinctive morphological characteristics, including bristle-like polar appendages on both ends of the ascospores and the absence of a gelatinous sheath. Konta et al. (2021) transferred *Endocalyx* from Apiosporaceae (Amphisphaeriales) to Cainiaceae based on morphology and multigene phylogeny. Li et al. (2022) revisited the monospecific genus *Alishanica*, synonymizing it under *Arecophila* through comprehensive molecular and morphological analyses. Recently, Han et al. (2024) introduced *Paramphibambusa* as a distinct basal lineage within Cainiaceae, characterized morphologically by a prominent long-necked ostiole and ascospores lacking longitudinal ornamentations. Currently, eight genera are recognized in Cainiaceae (Hyde et al. 2024).

Hyde (1996) introduced *Arecophila* with *Ar. gulubiicola* as the type species. Initially, due to its unitunicate, cylindrical asci with a J+ apical ring and brown, 2-celled ascospores, it was placed within Amphisphaeriaceae. Kang et al. (1999) subsequently transferred *Arecophila* into Cainiaceae. Smith et al. (2003) further supported this placement through single and combined sequence analyses of LSU and SSU loci. Continued phylogenetic studies by Jeewon et al. (2003); Senanayake et al. (2015); Hyde et al. (2020); Samarakoon et al. (2022); Li et al. (2022) and most recently Han et al. (2024); Hyde et al. (2024); Habib et al. (2025), and Sun et al. (2025) have consistently supported the inclusion of *Arecophila* within Cainiaceae. Currently, 26 species epithets are listed under *Arecophila* in Species Fungorum (2025).

*Amphibambusa* was introduced by Liu et al. (2015) with *Am. bambusicola* as its type species. This genus is characterized by immersed, light brown, coriaceous, globose to subglobose, uni-loculate ascomata, with papillate ostiole, located by a white margin, unitunicate, cylindrical, asci with a J+ apical ring, subapical ring and fusiform, hyaline, 1-septate, longitudinally striated ascospores, encased in a gelatinous sheath (Liu et al. 2015; Jiang et al. 2021). Currently, seven species are recognized within this genus, *viz*., *Am. aquatica*, *Am. aureae*, *Am. bambusicola*, *Am. cerosissimae*, *Am. guangxiensis*, *Am. hongheensis* and *Am. subbambusicola* (Species Fungorum 2025). While three of these (*Am. aureae*, *Am. bambusicola*, and *Am. hongheensis*) inhabit terrestrial bamboo culms, *Am. aquatica* is known from submerged decaying wood in freshwater ecosystems (Liu et al. 2015; Jiang et al. 2021; Manawasinghe et al. 2025; Zhou et al. 2024). However, the asexual morph of *Amphibambusa* remains undetermined.

Genera within Cainiaceae are predominantly associated with Poaceae and Arecaceae hosts (Liu et al. 2015; Jiang et al. 2021; Konta et al. 2021; Li et al. 2022; Han et al. 2024; Zhou et al. 2024). However, certain genera, such as *Seynesia* and *Longiappendispora*, exhibit associations with a broader range of host plant families (Mapook et al. 2020). Notably, *Arecophila*, all species except *Ar. deutziae* and *Ar. foveata* are exclusively associated with Poaceae and Arecaceae (Wang et al. 2004). *Amphibambusa* species similarly show a strong association with Poaceae, with the notable exception of *Am. aquatica* (Manawasinghe et al. 2025).

Grassland ecosystems provide significant opportunities for investigating fungal diversity due to their complex interactions (Murphy et al. 2016; Gao et al. 2024a, b). In the grassland biome, several living organisms, such as insects, herbivorous mammals, and fungi (saprobic, pathogenic, and symbiotic), play essential roles in maintaining biodiversity and ecosystem stability (Karunarathna et al. 2021). Studies from Sichuan and Yunnan indicate that grassland habitats support diverse fungal assemblages across multiple host groups and substrates, reinforcing these provinces as important regions for documenting grassland fungal diversity in China (Dissanayake et al. 2024a, b; Gao et al. 2024a, b, 2025; Wanasinghe and Maharachchikumbura 2023; Wanasinghe et al. 2025). Among grassland hosts, bamboo is notably recognized for supporting extensive fungal diversity (Jiang et al. 2022). Southwest China, especially in Yunnan Province is considered as a hotspot for bamboo diversity in China (Jiang et al. 2022).

In the present study, we collected microfungi associated with bamboo and other Poaceae hosts across various locations in Sichuan and Yunnan Provinces. Preliminary morphological examinations and phylogenetic analyses revealed five fungal taxa belonging to Cainiaceae. Our objectives are to: 1) characterize and document the morphological features of the collected samples; 2) conduct comprehensive phylogenetic analyses based on multigene sequence data to resolve their taxonomic positions; 3) introduce and formally describe two new species and report one known species in the genus *Arecophila*, one new species in *Amphibambusa* and one novel species within *Longiappendispora*; and 4) discuss the phylogenetic relationships and close affiliations among these newly introduced and previously known species within Cainiaceae. Detailed morphological descriptions, phylogenetic analyses and discussions are presented herein.

## Materials and methods

### Sample collection and morphological observations

Between 2021 and 2023, we collected Poaceae samples from five habitats in Sichuan and Yunnan, China. In Sichuan, sampling took place in Dujiangyan City (~1065 m a.s.l.) within mixed-species woodland dominated by bamboo under a subtropical climate, with collections made in winter. In Yunnan, we focused on plateau vegetation across an elevation gradient and sampled in both the rainy and dry seasons at four sites: Honghe (Mile City, ~1950m a.s.l.; mixed forest; subtropical climate; summer collections); Shangri-La (Deqen; ski area, ~2250 m a.s.l.; mixed forest; subtropical highland climate; summer collections); and two sites in Xishuangbanna *viz*. Yinchang Mountain (~1188 m a.s.l.; natural bamboo forest; tropical climate; winter collections) and Mengla County (~776 m a.s.l.; mixed forest; tropical climate; winter collections). The important collection information was reported as described by Rathnayaka et al. (2024). Specimens were stored in plastic Ziplock bags and transferred to the mycology laboratory at the Kunming institute of Botany (Kunming, China) for further examination. Morphological studies were conducted using a Motic SMZ 168 Series stereomicroscope. For the observation of morphological characteristics, we used the technique of hand sectioning of sporocarps. This procedure involved carefully slicing the sporocarps and placing the resulting sections onto water-mounted glass slides. Melzer’s reagent and Indian ink were used as needed. Microscopic photography was conducted using a Nikon ECLIPSE Ni-U complex microscope fitted with a Canon EOS 600D camera. Measurements were made with the Tarosoft (R) Image Frame Work program. We used Adobe Photoshop CS6 to process and refine the images that would be incorporated into our figures, making them more illustrative and comprehensible. Single spore isolation on these sample were done according to the standardized procedure described by Senanayake et al. (2020). Type specimens (holotype and isotype) were deposited in the Herbarium of Cryptogams, Kunming Institute of Botany, Chinese Academy of Sciences (KUN-HKAS), Kunming, China. The living cultures were deposited at the Kunming Institute of Botany Culture Collection (KUNCC), Kunming, China. The new taxon was registered in MycoBank (https:/www.mycobank.org).

### DNA extraction, PCR amplification and sequencing

DNA extraction, PCR amplification and sequencing were carried out following the methods described in Dissanayake et al. (2024a, b). For DNA extraction, mycelia were cultured from each fungal isolate using potato dextrose agar (PDA; potato extract 4 g/L [equivalent to 200g of infusion from potatoes], glucose 20 g/L, agar 15 g/L). The cultures were grown for 3–4 weeks under room conditions at 28 °C. Total genomic DNA was extracted from the thriving cultures, focusing on approximately 150±50 mg of axenic mycelium. This mycelium was scraped from the periphery of the expanding culture to ensure its purity. The Biospin Fungus Genomic DNA Extraction Kit-BSC14S1 (produced by BioFlux, P.R. China) was used for the DNA extraction, following the manufacturer’s guidelines.

However, fungi failed to grow on culture media (HKAS 130468, HKAS 130469, HKAS 130459, HKAS 130460), an alternative approach for DNA extraction was implemented. DNA was extracted directly from the whole fruiting bodies. This procedure adhered to the protocol described by Wanasinghe et al. (2025). Genomic DNA was amplified by polymerase chain reaction (PCR). Four phylogenetic markers, internal transcribed spacer (ITS) and large-subunit ribosomal RNA (LSU) were amplified using primer pairs ITS4/ITS5 (White et al. 1990) and LR5/LR0R (Vilgalys and Hester 1990) respectively. Amplification conditions were performed according to Han et al. (2024) and Zhou et al. (2024). The purified PCR fragments were sent to a commercial sequencing provider (BGI, Ltd Shenzhen, P.R. China). The nucleotide sequence data acquired were deposited in GenBank for further reference and use.

### Phylogenetic analyses

Newly generated sequences were subjected to BLAST search in the NCBI GenBank database and sequences of closely related taxa were downloaded. Phylogenetic analysis was performed using ITS and LSU sequences (Table 1). Multiple alignments, including both consensus and reference sequences, were generated using MAFFT v. 7 (Katoh et al. 2019) and manually refined using BioEdit v. 7.0.5.2 (Hall 1999). The individual datasets were combined into a concatenated dataset and further refined in BioEdit. Both combined and individual datasets were subjected to maximum likelihood (ML) and Bayesian inference (BI) analyses. The best-fit substitution models were evaluated using MrModeltest v. 2.3 (Nylander 2004) with the Akaike Information Criterion (AIC) as the selection criteria executed in PAUP v. 4.0b10 (Swofford 2003). Phylogenetic analyses using both ML and BI approaches were conducted via the CIPRES Science Gateway (Miller et al. 2010). For ML analyses, RAxML-HPC2 on XSEDE v. 8.2.10 (Stamatakis et al. 2008; Stamatakis 2014) was used, applying the GTR+I+G model with 1000 bootstrap repetitions. Bayesian analyses were carried out using MrBayes on ACCESS (Miller et al. 2010), implementing the GTR+I+G substitution model. The MCMC algorithm was run for 2 million generations, with trees sampled every 1,000 generations. The run terminated automatically once the average standard deviation of split frequencies fell below 0.01, and the first 25% of trees were discarded as burn-in. ML bootstrap values (MLBS) ≥70% and Bayesian posterior probabilities (BYPP) ≥0.95 are reported above the corresponding branches in the phylogenetic trees. Phylogenetic tree reliability was assessed based on MLBS and BYPP. Nodes with MLBS ≥90% and BYPP ≥0.95 were considered robustly supported, nodes with MLBS between 75–89% and BYPP between 0.90–0.94 were considered strongly supported and nodes with MLBS <75% and BYPP <0.90 were considered weakly or not significantly supported (Hillis and Bull 1993; Alfaro et al. 2003; Soltis and Soltis 2003). The phylogram was visualized using the FigTree v1.4.0 program (Rambaut and Drummond 2012) and annotated using Microsoft PowerPoint (2019).

**Table 1.** Taxa (strains and sequences) used in the phylogenetic analyses.

Species name	Strain no.	GenBank accession no.		Reference
		ITS	LSU	
*Amphibambusa aquatica*	MFLUCC 18-1046 ^T^	PP584660	PP584659	Manawasinghe et al. (2024)
*Am. aureae*	GMB4550 ^T^	PQ066508	PQ066514	Zhou et al. (2024)
*Am. aureae*	GMB4561	PQ066509	PQ066515	Zhou et al. (2024)
*Am. bambusicola*	GMB5602 ^T^	PQ884689	PQ885401	Liu et al. (2025)
*Am. bambusicola*	GMB5608	PQ884690	PQ885402	Liu et al. (2025)
*Am. bambusicola*	MFLUCC 11-0617	KP744433	KP744474	Liu et al. (2015)
*Am. cerosissimae*	GMB5603 ^T^	PQ884693	PQ885405	Liu et al. (2025)
*Am. cerosissimae*	GMB5613	PQ884694	PQ885406	Liu et al. (2025)
*Am. hongheensis*	KUN-HKAS 112723 ^T^	PQ066511	MW892969	Jiang et al. (2021)
*Am. hongheensis*	KUNMCC 20-0334	PQ066512	MW892970	Jiang et al. (2021)
***Am. hyalinospora***	**KUNCC23-15547 ^T^**	**PX527199**	**PX527209**	**This study**
***Am. hyalinospora***	**KUNCC23-15548**	**PX527200**	**PX527210**	**This study**
*Am. subbambusicola*	GMB5606 ^T^	PQ884691	PQ885403	Liu et al. (2025)
*Am. subbambusicola*	GMB5618	PQ884692	PQ885404	Liu et al. (2025)
*Arecophila amphibambusina*	GMB6259 ^T^	PQ874066	PQ860514	Habib et al. (2025)
*Ar. amphibambusina*	GMB6260	PQ874067	PQ860515	Habib et al. (2025)
*Ar. australis*	GZUCC0112 ^T^	PQ066513	MT742133	Li et al. (2022)
*Ar. australis*	GZUCC0124	PQ066514	MT742132	Li et al. (2022)
*Ar. bambusae*	HKUCC 4794	–	AF452038	Kang et al. (1999)
*Ar. chinensis*	GMB6217 ^T^	PQ874007	PQ860452	Habib et al. (2025)
*Ar. chinensis*	GMB6218	PQ874008	PQ860453	Habib et al. (2025)
*Ar. clypeata*	GZUCC0110 ^T^	MT742129	MT742136	Li et al. (2022)
*Ar. clypeata*	GZUCC0127	MT742128	MT742135	Li et al. (2022)
***Ar. clypeata***	**KUNCC23-15549**	**PX527203**	**PX527213**	**This study**
***Ar. clypeata***	**KUNCC23-15550**	**PX527204**	**PX527214**	**This study**
*Ar. gaofengensis*	GMB454 ^T^	PQ066512	PQ066516	Zhou et al. (2024)
*Ar. gaofengensis*	GMB4559	PQ066513	PQ066517	Zhou et al. (2024)
*Ar. guizhouensis*	GMB1138 ^T^	PQ884695	PQ885407	Liu et al. (2025)
*Ar. guizhouensis*	GMB5614	PQ884696	PQ885408	Liu et al. (2025)
*Ar. maolanensis*	GUCC 24-0116 ^T^	PQ568137	PQ569318	Sun et al. (2025)
***Ar. menglaensis***	**KUNCC23-15551 ^T^**	**PX527201**	**PX527211**	**This study**
***Ar. menglaensis***	**KUNCC23-15552**	**PX527202**	**PX527212**	**This study**
*Ar. miscanthi*	MFLU 19-2333 ^T^	NR_171235	MK503827	Hyde et al. (2020)
*Ar. muroiana*	GZUCC0122 ^T^	MT742127	MT742134	Li et al. (2022)
***Ar. sichuanensis***	**HKAS 130468 ^T^**	**PX527197**	**PX527207**	**This study**
***Ar. sichuanensis***	**HKAS 130469**	**PX527198**	**PX527208**	**This study**
*Ar. subguizhouensis*	GMB5617 ^T^	PQ884697	PQ885409	Liu et al. (2025)
*Ar. subguizhouensis*	GMB5610	PQ884698	PQ885410	Liu et al. (2025)
*Ar. xishuangbannaensis*	ZHKU 23-0280	OR995737	OR995744	Han et al. (2024)
*Ar. xishuangbannaensis*	GMB-W1283 ^T^	OR995736	OR995743	Han et al. (2024)
*Ar. yunnanensis*	GUCC 24-0117 ^T^	–	PQ569319	Sun et al. (2025)
*Ar. zhaotongensis*	GMBCC1145 ^T^	OR995740	OR995747	Han et al. (2024)
*Ar. zhaotongensis*	ZHKU 23-0260	OR995738	OR995745	Han et al. (2024)
*Ar. zhaotongensis*	ZHKU 23-0259	OR995735	OR995742	Han et al. (2024)
*Arecophila* sp.	HKUCC 6487	–	AF452039	Jeewon et al. (2003)
*Barrmaelia shangrilaensis*	HKAS 130280 ^T^	NR_198756	PP584818	Dissanayake et al. (2024)
*B. yunnanensis*	HKAS 130278 ^T^	NR_198757	PP584820	Dissanayake et al. (2024)
*Cainia anthoxanthi*	MFLUCC 15-0539 ^T^	NR138407	NG070382	Senanayake et al. (2015)
*C. daweishanensis*	GMB5619 ^T^	PQ884699	PQ885411	Liu et al. (2025)
*C. daweishanensis*	GMB5624	PQ884700	PQ885412	Liu et al. (2025)
*C. globosa*	MFLUCC 13-0663 ^T^	NR171724	KX822123	Hyde et al. (2016)
*C. graminis*	CBS:136.62	MH858123	MH869701	Vu et al. (2019)
*C. graminis*	MFLUCC 15-0540 ^T^	KR092793	KR092781	Senanayake et al. (2015)
*C. shilihetanensis*	GMB5612 ^T^	PQ884701	PQ885413	Liu et al. (2025)
*C. shilihetanensis*	GMB5622	PQ884702	PQ885414	Liu et al. (2025)
*Endocalyx cinctus*	NBRC 31306	MZ313191	MZ313152	Delgado et al. (2022)
*E. grossus*	JCM 5164 ^T^	MZ313160	MZ313138	Delgado et al. (2022)
*E. grossus*	JCM 5165	MZ313159	MZ313158	Delgado et al. (2022)
*E. indumentum*	JCM 5171 ^T^	MZ313153	MZ313161	Delgado et al. (2022)
*E. metroxyli*	MFLUCC 15-0723A	MT929162	MT929313	Konta et al. (2021)
*E. ptychospermatis*	ZHKUCC 21 0008 ^T^	MZ493352	OK569894	Phukhamsakda et al. (2022)
*Longiappendispora chromolaenae*	MFLUCC 17-1485 ^T^	MT214370	MT214464	Mapook et al. (2020)
***L. shangrilaensis***	**HKAS 130459 ^T^**	**PX527195**	**PX527205**	**This study**
***L. shangrilaensis***	**HKAS 130460**	**PX527196**	**PX527206**	**This study**
*Paramphibambusa bambusicola*	GMBCC1142 ^T^	OR995739	OR995746	Han et al. (2024)
*P. bambusicola*	ZHKUCC 23-0976	OR995741	OR995748	Han et al. (2024)
*Seynesia erumpens*	SMH 1291 (F)	–	AF279410	Bhattacharya et al. (2000)

Sequences obtained in this study are shown in bold. Type specimen or ex-type strain marked with ‘^T^’; unavailable data or sequences are marked with ‘–’.

## Results

### Phylogenetic analysis

**Figure 1.**
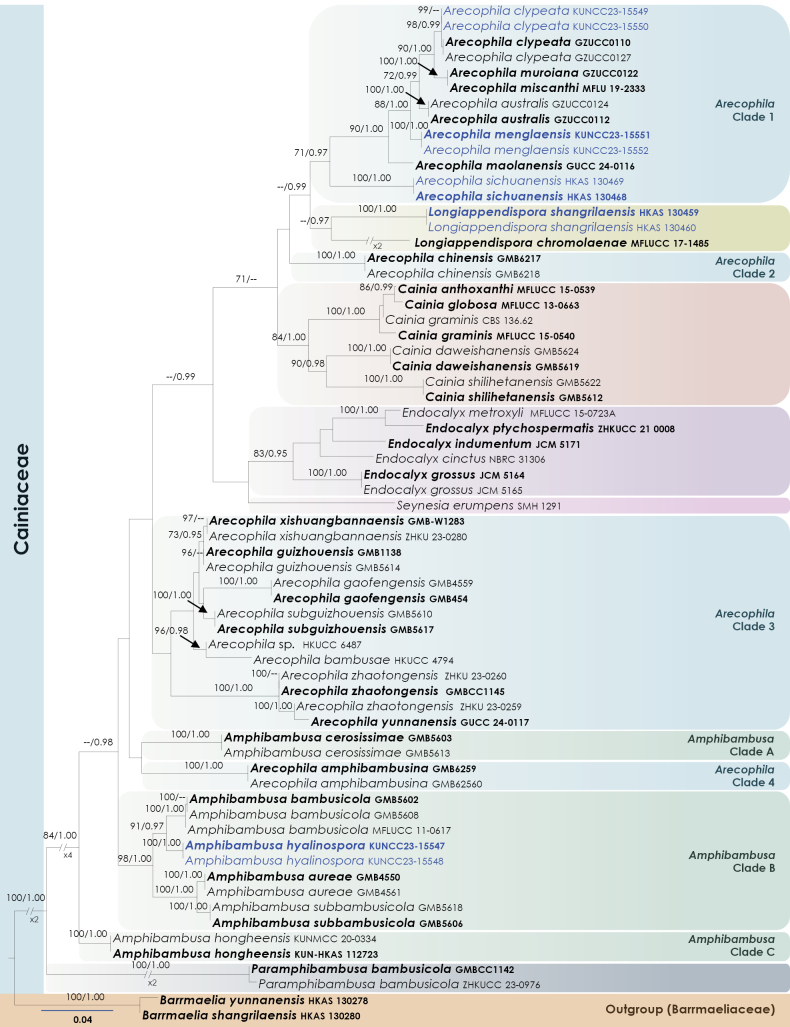
Maximum Likelihood tree inferred from the concatenated dataset of partial LSU and ITS sequences representing members of Cainiaceae. The phylogeny is rooted with *Barrmaelia shangrilaensis* (HKAS 130280) and *B. yunnanensis* (HKAS 130278). In the resulting phylograms, sequences generated in this study are highlighted in blue. Species names given in bold are ex-type strains.

To investigate the phylogenetic relationships of our new strains within Cainiaceae, phylogenetic analyses were conducted based on the combined LSU and ITS DNA sequences of 66 representatives of Cainiaceae taxa, along with the outgroup taxa of *Barrmaelia shangrilaensis* (HKAS 130280) and *B. yunnanensis* (HKAS 13027) from Barrmaeliaceae. The full dataset comprised 1334 characters including gaps (LSU= 851 characters, ITS 480). The RAxML analysis of the combined dataset yielded a best-scoring tree with a final ML optimization likelihood value of -8268.468294. The matrix contained 437 distinct alignment patterns, with 6.18% undetermined characters or gaps. Parameters for the GTR model of the combined amplicons were as follows: Estimated base frequencies; A =0.250774, C =0.233520, G =0.283267, T =0.232440; substitution rates AC =2.289088, AG = 4.191579, AT =4.079836, CG =2.416527, CT =10.785761, GT =1.000000; proportion of invariable sites I = 0.557203; and gamma distribution shape parameter α =0.504249. The Bayesian analysis ran 706000 generations before the average standard deviation for split frequencies reached below 0.01 (0.009987). The analyses generated 7061 trees, from which we sampled 5296 trees after discarding the first 25% as burn-in. The BI and ML trees were not in conflict; the ML tree is presented in Fig. 1.

In our phylogenetic analyses, we used all available sequence data for *Amphibambusa*, *Arecophila*, *Cainia*, *Endocalyx*, *Longiappendispora*, *Paramphibambusa* and *Seynesia*. In both ML and BI analyses, *Amphibambusa* and *Arecophila* strains were not monophyletic and topology is similar to previous studies (e.g. Han et al. 2024; Zhou et al. 2024; Habib et al. 2025).

*Amphibambusa* strains formed three distinct monophyletic clades (Fig. 1). Clade A comprised two strains of *Am. cerosissimae* (GMB5603, GMB5613), with robust support values (100% MLBS, 1.00 BYPP).

Clade B included strains of *A. aureae* (GMB4550, GMB4561), *Am. bambusicola* (GMB5602, GMB5608, MFLUCC 11-0617), two newly collected isolates introduced here as *Am. hyalinospora* sp. nov. (KUNCC23-15547, KUNCC23-15548) and *Am. subbambusicola* (GMB5606, GMB5618) with 98% MLBS and 1.00 BYPP support values. Clade C was basal within the family, represented by *Am. hongheensis* (KUNMCC 20-0334, KUN-HKAS 112723).

*Arecophila* strains formed four distinct lineages. Clade 1 consisted of *Ar. australis* (GZUCC0112, GZUCC0124), *Ar. clypeata* (GZUCC0110, GZUCC0127), *Ar. maolanensis* (GUCC 24-0116), *Ar. miscanthi* (MFLU 19-2333) and *Ar. muroiana* (GZUCC0122). Within Clade 1, two new isolates (KUNCC23-15549, KUNCC23-15550) grouped with *Ar. clypeata*. New isolates of two species introduced here as new: *Ar. menglaensis* (KUNCC23-15551, KUNCC23-15552) and *Ar. sichuanensis* (HKAS 130468, HKAS 130469) clustered in distinct subclades within Clade 1. Clade 3, lacking significant support, comprised *Ar. bambusae* (HKUCC 4794), *Ar. gaofengensis* (GMB454, GMB4559), *Ar. guizhouensis* (GMB1138, GMB5614), *Arecophila* sp. (HKUCC 6487), *Ar. subguizhouensis* (GMB5610, GMB5617), *Ar. xishuangbannaensis* (GMB-W1283, ZHKU 23-0280), *Ar. yunnanensis* (GUCC 24-0117) and *Ar. zhaotongensis* (GMBCC1145, ZHKU 23-0259, ZHKU 23-0260). *Arecophila amphibambusina* (GMB6259, GMB6260) formed Clade 4, closely affiliated with *Amphibambusa cerosissimae*.

*Cainia* strains formed a monophyletic group with high support values (84% MLBS, 1.00 BYPP). *Endocalyx* strains similarly grouped monophyletically, with moderate support (83% MLBS, 0.95 BYPP). *Longiappendispora chromolaenae* (MFLUCC 17-1485) and our two new isolates (HKAS 130459, HKAS 130460), introduced here as *L. shangrilaensis* sp. nov., formed a monophyletic clade positioned between *Arecophila* Clades 1 and 2. *Paramphibambusa bambusicola* (GMBCC1142, ZHKUCC 23-0976) represented the most basal lineage within the family. *Seynesia erumpens* (SMH 1291) nested as a sister lineage to *Endocalyx*.

### Taxonomy

**Xylariales Nannf., Nova Acta Regiae Soc. Sci. Upsal. Ser. 4, 8 (2): 66 (1932)**

**Cainiaceae J.C. Krug, Sydowia 30 (1–6): 123 (1978)**

#### Amphibambusa

Taxon classificationFungiAmphisphaerialesAmphisphaeriaceae

D.Q. Dai & K.D. Hyde, Fungal Diversity 72: 7 (2015)

D98296DA-1E97-5AB0-AEC5-0A374C05EAA1

##### Notes.

Species of *Amphibambusa* are saprobic fungi that primarily colonize dead plant materials, especially culms of Bambusoideae members and other monocots. They also occur on submerged decaying wood in freshwater, indicating a broader ecological niche that includes both terrestrial and aquatic environments. The genus is known from tropical Asia, particularly China and Thailand (Hyde et al. 2020; Zhou et al. 2024; Liu et al. 2025). Seven species are currently accepted in Index Fungorum (2025) and with the addition reported here, the total increases to eight. Our record represents the fifth *Amphibambusa* species documented from Yunnan Province, China.

#### Amphibambusa hyalinospora

Taxon classificationFungiAmphisphaerialesAmphisphaeriaceae

L.S. Dissan. & Wanas. sp. nov.

E09A9A12-C5F0-544F-899F-2CB2F8455ADF

861156

Fig. 2

##### Etymology.

The specific epithet refers to the hyaline ascospores.

##### Holotype.

HKAS 149994.

##### Description.

***Saprobic*** on the surface of decaying bamboo culms. **Sexual morph. *Ascomata*** 590–670 × 400–500 μm (x̄ = 645 × 467 μm, n = 10), solitary, scattered, immersed under the host epidermis, visible as black pointed spots on the host surface, coriaceous, ostiole at the center. ***Peridium*** 25–35 μm (x̄ = 30 μm, n = 10), multi-layered, outer layer comprising blackish-brown, composed of thick-walled cells of ***textura angularis***, inner layer composed of pale brown to hyaline, thin-walled cells of ***textura angularis***, tightly arranged. ***Paraphyses*** 5–9 μm (x̄ = 6.9 μm, n = 20) wide, intermingled among asci, cylindrical, septate, thin-wall, hyaline, guttulate. ***Asci*** 180–260 × 13–20 μm (x̄ = 222 × 17.2 μm, n = 20), 8-spored, unitunicate, cylindrical to elongate fusiform, wider in the middle, tapering towards both ends, with rounded end, short-pedicellate, J+ apical ring in Melzer’s reagent. ***Ascospores*** 80–110 × 6–10 μm (x̄ = 95 × 8 μm, n = 20), overlapping, 1–2 seriate, hyaline, fusiform, 1-septate at the center, pointed at both ends, with hyaline round spots when mature, with longitudinal striation along the entire length of the ascospore, with thin-gelatinous sheath. **Asexual morph**. Unknown.

**Figure 2.**
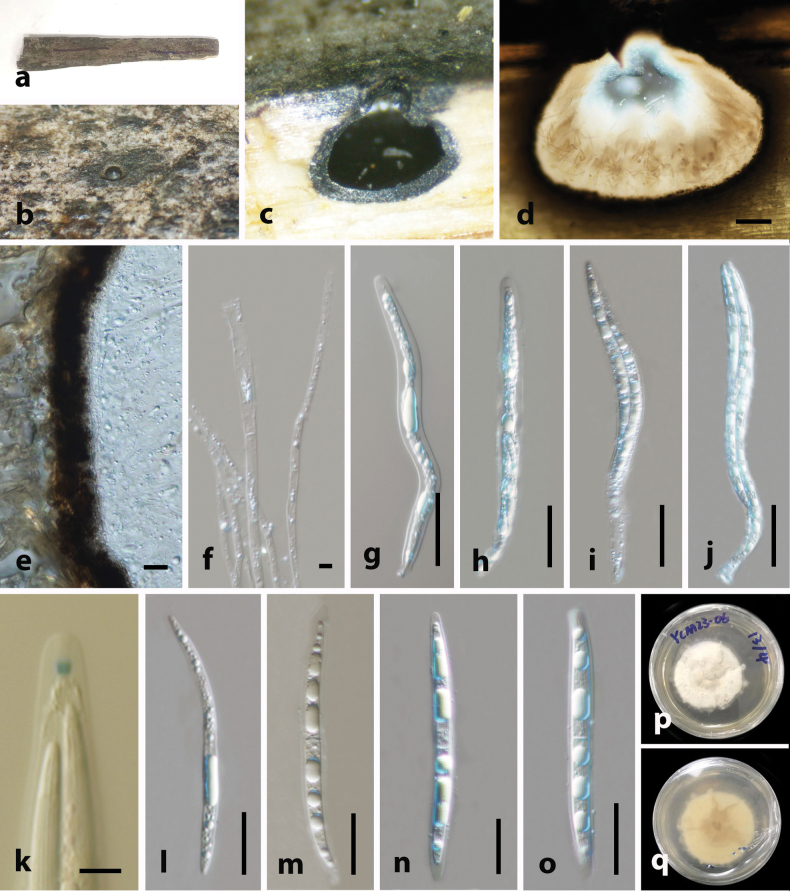
*Amphibambusa hyalinospora* (holotype HKAS 149994). **a, b**. Ascomata on the host surface; **c, d**. Section of ascoma; **e**. Peridium; **f**. Paraphyses; **g–j**. Asci; **k**. Apical ring bluing in Melzer’s reagent; **l–o**. Ascospores; **p**. Upper view of the one-week-old colony on PDA; **q**. Reverse view. Scale bars: 50 μm (**d**); 10 μm (**e, f**); 50 μm (**g–j**); 20 μm (**k–o**).

##### Culture characters.

Colonies on PDA, reaching 25–30 mm diameter after one week at 25 °C, white, cottony flat, low, dense, with uneven margin. Reverse yellowish-brown at the center and yellowish-white edges.

##### Material examined.

• China, Yunnan Province, Xishuangbanna, Yinchang Mountain (21.968333°N, 101.1975°E, 1188 m a.s.l.), on dead culm of bamboo, 16 February 2023, L.S. Dissanayake, XTBGYCM23-06 (HKAS 149994, **holotype**), ex-type KUNCC23-15547. *ibid*. XTBGYCM23-06A (HKAS 149995, isotype), ex-isotype, KUNCC23-15548.

##### Note.

Phylogenetic analyses based on multi-locus sequence data reveal that *Am. hyalinospora* is closely related to *Am. bambusicola* (GMB5602: ex-type, GMB5608 and MFLUCC 11-0617), with 95% MLBP and 0.97BYPP support values (Fig. 1). *Amphibambusa hyalinospora* distinguish from *Am. bambusicola* having comparatively longer ascospores (95 × 8 μm), constricted at the septum with thin sheath while *Am. bambusicola* has shorter ascospores (26.6 × 5.7 μm) that are deeply constricted at the septum with thick gelatinous sheath (10 μm) (Liu et al. 2025). A comparison of nucleotide differences (without gaps) between these two species showed 14/451 (3.1%) differences in the ITS region and 9/848 (1.06%) differences in the LSU region. Thus, the combined phylogenetic, morphological and sequence divergence evidence supports recognition of *Amphibambusa hyalinospora* as a distinct species closely related to *Am. bambusicola*.

#### Arecophila

Taxon classificationFungiXylarialesCainiaceae

K.D. Hyde, Nova Hedwigia 63: 82 (1996)

A7E7942A-9704-5D0F-B2AE-BFD3E6D24A92

##### Notes.

*Arecophila* species are typically saprobes on decaying or dead substrates, including wood, culms and leaves, and they are widely distributed across tropical, subtropical and temperate regions (Hyde et al. 2020; Li et al. 2022). Currently, 26 epithets are recorded in Index Fungorum (2025). The present study adds two novel taxa to the genus, increasing the total from 26 to 28.

#### Arecophila clypeata

Taxon classificationFungiXylarialesCainiaceae

Q.R. Li, J.C. Kang & K.D. Hyde MycoKeys 88: 135 (2022)

445C204B-CE48-5FA6-A25E-34A70AD4DAFD

836167

Fig. 3

##### Description.

***Saprobic*** on dead culms of bamboo hosts. **Sexual morph. *Ascomata*** 200–260 μm × 300–325 μm (x̄ = 230 × 320 μm, n = 5), immersed under a black clypeus, slightly raised, dome-shaped areas, blackened, scattered or gregarious, globose to subglobose, uniloculate or multiloculate with a central, erumpent, cone-shaped papillae in vertical section. ***Ostioles*** 60–65 μm × 45–55 μm (x̄ = 63 × 51 μm, n = 5), papillate, with hyaline periphyses, black peridium. ***Peridium*** 30–40 μm wide (x̄ = 35 μm, n = 5), comprising several layers, outer layer brown, composed of thick-walled cells of ***textura angularis*** and inner layer hyaline, arrange with cells of ***textura prismatica***. ***Paraphyses*** 2–5 μm wide (x̄ = 3 μm, n = 10), hyaline, unbranched, cellular, aseptate. ***Asci*** 150–230 × 11–13 μm (x̄ = 185 × 12 μm, n = 20), 8-spored, cellular, long-cylindrical, short-pedicellate, apically rounded, with trapezoidal, J+, apical ring. ***Ascospores*** 18–25 × 6–10 μm (x̄ = 22 × 8 μm, n = 5), overlapping, uniseriate, ellipsoidal, 2-celled, guttulate, hyaline to golden brown, constricted at the septum, weakly sulcate striations along the entire length of mature spore, surrounded by a thick mucilaginous sheath in immature and thin mucilaginous sheath in mature. **Asexual morph**. Unknown.

**Figure 3.**
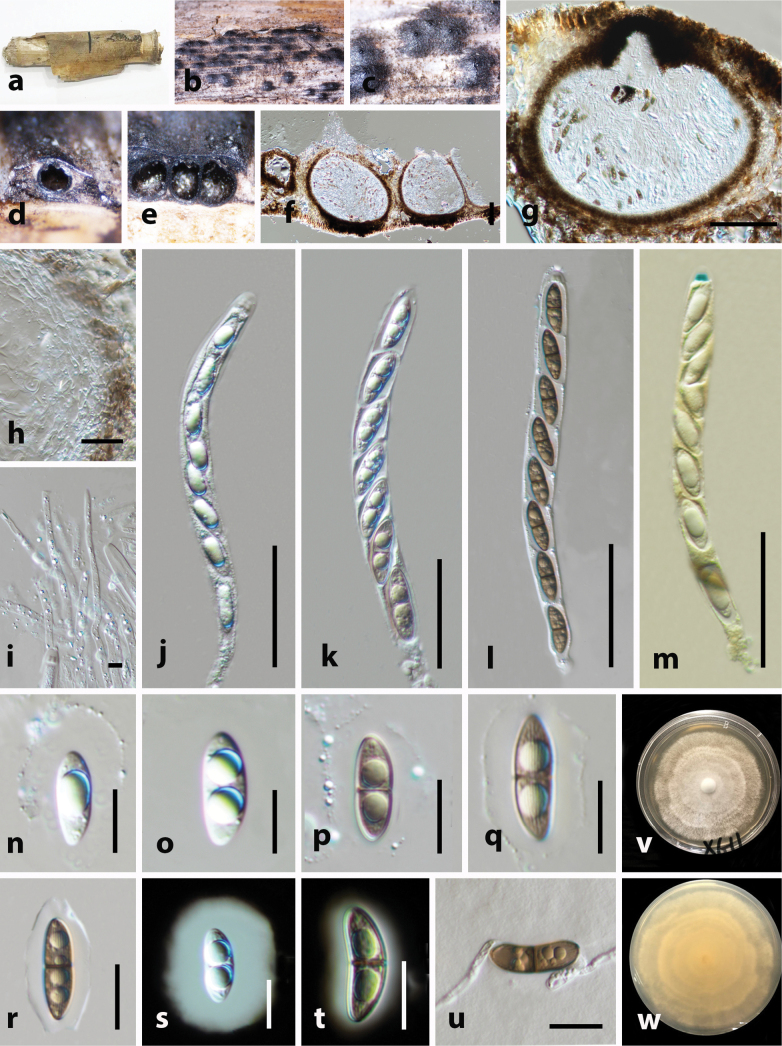
*Arecophila clypeata* (HKAS 130466). **a**. Substrate; **b, c**. Ascomata on the host surface; **d–g**. Section of ascoma; **h**. Peridium; **i**. Paraphyses; **j–m**. Asci; **m**. Apical ring bluing in Melzer’s reagent; **n–u**. Ascospores; **s, t**. Immature and mature ascospores stained with Indian ink (**s**. Immature; **t**. Mature); **u**. Germinated ascospore; v. Upper view of the one-week-old colony on PDA; **w**. Reverse view. Scale bars: 100 μm (**f, g**); 20 μm (**h, n–u**); 5 μm (**i**); 50 μm (**j–m**); 10 μm (**k–q**).

##### Culture characteristics.

Colonies on PDA, reaching 25–30 mm diameter after one week at 25 °C, white, cottony flat, low, dense, uneven margin. Reverse light brownish at the center and whitish edges.

##### Material examined.

• China, Yunnan Province, Mile City, Xiyizhen (24.4930°N, 103.3050°E, 1950m a.s.l.), on dead culms of a bamboo host, 29 May 2021, Gao Yin, GYXG22-11 (HKAS 130466), living culture, KUNCC23-15549. *ibid*. GYXG22-11A (HKAS 130467), living culture, KUNCC23-15550.

##### Additional GenBank accession numbers.

*btub* = PX716850, PX716851.

##### Note.

Our newly collected strains (HKAS 130466and HKAS 130467) are morphologically identical to *Arecophila clypeata* by their globose to subglobose, cone-shaped papillate ascomata, hyaline, unbranched paraphyses, long-cylindrical, short-pedicellate asci with J+, apical ring and 1-septate, pale brown to brown, ascospores surrounded by a mucilaginous sheath (Li et al. 2022). However, we noted aseptate paraphyses in our collection whereas septate paraphyses in the holotype (GZUCC0110) (Li et al. 2022). According to the phylogenetic analysis, our two strains are nested together with *Arecophila clypeata* (GZUCC0110 and GZUCC0127) without significant statistical support. Comparison of nucleotide differences between these isolates and the *Ar. clypeata* are not showing any differences. Therefore, we recognize our new collections as conspecific with *Arecophila clypeata*.

#### Arecophila menglaensis

Taxon classificationFungiXylarialesCainiaceae

L.S. Dissan. & Wanas. sp. nov.

55D72B01-72F9-5A21-A96E-EF7F8369BF68

861157

Fig. 4

##### Etymology.

The specific epithet is derived from Mengla County, where the holotype was collected

**Figure 4.**
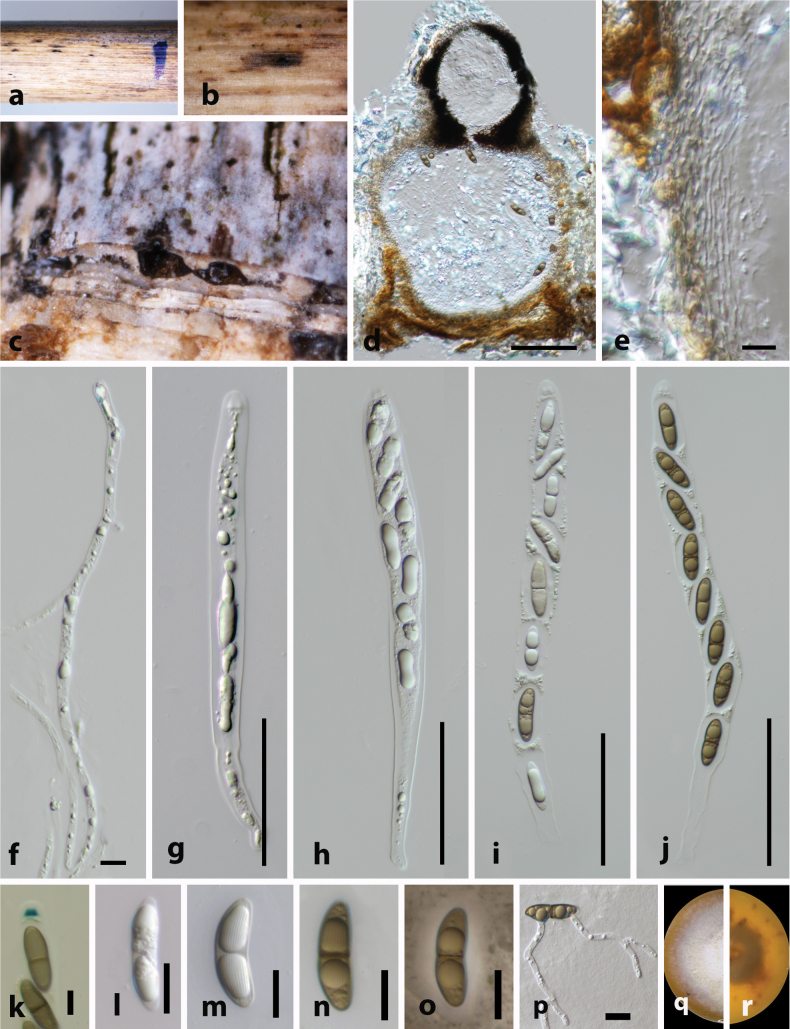
*Arecophila menglaensis* (holotype HKAS 130461). **a, b**. Ascomata on the host surface; **c, d**. Section of ascoma; **e**. Peridium; **f**. Paraphyses; **g–j**. Asci; **k**. Apical ring bluing in Melzer’s reagent; **l–o**. Ascospores: **o**. Ascospore stained with Indian ink; **p**. Germinated ascospore; **q**. Upper view of the one-week-old colony on PDA; **r**. Reverse view. Scale bars: 100 μm (**d**); 10 μm (**e**); 10 μm (**f, k**); 50 μm (**g–j**); 10 μm (**l–p**).

##### Holotype.

HKAS 130461.

##### Description.

***Saprobic*** on dead culm of Poaceae host. **Sexual morph. *Ascomata*** 190–320 μm × 200–390 μm (x̄ = 257 × 285 μm, n = 5), immersed, solitary, slightly raised, blackened, scattered or gregarious, globose to subglobose, uniloculate, with a central, erumpent, papillae in vertical section. ***Ostioles*** 90–110 μm × 140–170 μm (x̄= 103 × 156 μm, n = 5), papillate, with hyaline periphyses, black peridium. ***Peridium*** 25–40 μm wide (x̄ = 30 μm, n = 5), comprising several layers, outer layer brown, composed of thick-walled cells of ***textura angularis*** and inner layer hyaline, arrange with cells of ***textura prismatica***. ***Paraphyses*** 3–5 μm wide (x̄ = 4 μm, n = 15), hyaline, unbranched, aseptate. ***Asci*** 125–190 μm × 15–20 μm (x̄ = 165 × 17 μm, n = 15), 8-spored, unitunicate, long-cylindrical, short-pedicellate, apically rounded, with trapezoidal, J+, apical ring. ***Ascospores*** 20–30 × 6–9 μm (x̄ = 24.2 × 7.2 μm, n = 20), overlapping, uniseriate, ellipsoidal, 2-celled, guttulate, hyaline to golden brown, constricted at the septum, weakly sulcate striations along the entire length of mature spore, surrounded by a thin mucilaginous sheath. **Asexual morph**. Undetermined.

##### Culture characteristics.

Colonies on PDA, reaching 25–30 mm diameter after one week at 25 °C, white, cottony, flat, low, dense, with uneven margin. Reverse brownish at the center and yellowish-brown edges.

##### Material examined.

• China, Yunnan Province, Mengla, Xishuangbanna (21.580788°N, 101.434776°E, 776 m a.s.l.), on dead culm of Poaceae host, 29 January 2023, L. S. Dissanayake, QML23_22 (HKAS 130461, **holotype**), ex-type, KUNCC23-15551. *ibid*. QML23_22A (HKAS 130462, isotype), KUNCC23-15552.

##### Note.

*Arecophila menglaensis* nested as a basal lineage of a monophyletic sister clade that included *Ar. clypeata* (KUNCC 23-15549, KUNCC 23-15550, GZUCC0110, GZUCC0127), *Ar. muroiana* (GZUCC0122), *Ar. miscanthi* (MFLU 19-2333) and *Ar. australis* (GZUCC0124 and GZUCC0112). However, *Arecophila menglaensis* can be distinguished from the three related species by its smaller ascomata (257 × 285 μm) and the absence of a clypeus. By comparison, *Ar. australis*, *Ar. clypeata* and *Ar. muroiana* have larger ascomata (495 × 325 μm, 403 × 323 μm, and 350–460 × 320–400 μm respectively), and a clypeus is present in *Ar. australis*, *Ar. clypeata* and *Ar. miscanthi* (Li et al. 2022). The phylogenetic placement of *Arecophila menglaensis* as a basal lineage within this clade, together with its diagnostic morphology (particularly the smaller ascomata and absence of a clypeus) supports its recognition as a distinct species within *Arecophila*.

#### Arecophila sichuanensis

Taxon classificationFungiXylarialesCainiaceae

L.S. Dissan. & Maharachch. sp. nov.

0992EE5E-EA58-5667-A64D-B01B3DE23773

861158

Fig. 5

##### Etymology.

The epithet is derived from Sichuan Province, where the holotype was collected.

##### Holotype.

HKAS 130468

##### Description.

***Saprobic*** on dead culm of Poaceae host. **Sexual morph. *Ascomata*** 210–280 μm × 300–380 μm (x̄ = 265 × 335 μm, n = 5), immersed under a black clypeus, slightly raised, blackened, scattered or gregarious, globose to subglobose, erumpent, papillate. ***Ostioles*** with hyaline periphyses, black peridium. ***Peridium*** 100–120 μm wide (x̄ = 110 μm, n = 5), comprising several layers, outer layer brown, composed of thick-walled cells of ***textura angularis*** and inner layer hyaline, arranged with ***textura angularis***. ***Paraphyses*** 4–6 μm wide (x̄ = 5 μm, n = 10), hyaline, unbranched, cellular, rarely septate. ***Asci*** 150–180 × 15–20 μm (x̄ = 165 × 18 μm, n = 20), 8-spored, long-cylindrical, short-pedicellate, apically rounded, with trapezoidal, J+, apical ring. ***Ascospores*** 20–25 × 6–10 μm (x̄ = 23 × 8 μm, n = 5), overlapping, uniseriate, ellipsoidal, 2-celled, guttulate, hyaline to brown, constricted at the septum, sulcate striations along the entire length of mature spore, surrounded by a thin mucilaginous sheath. **Asexual morph**. Undetermined.

**Figure 5.**
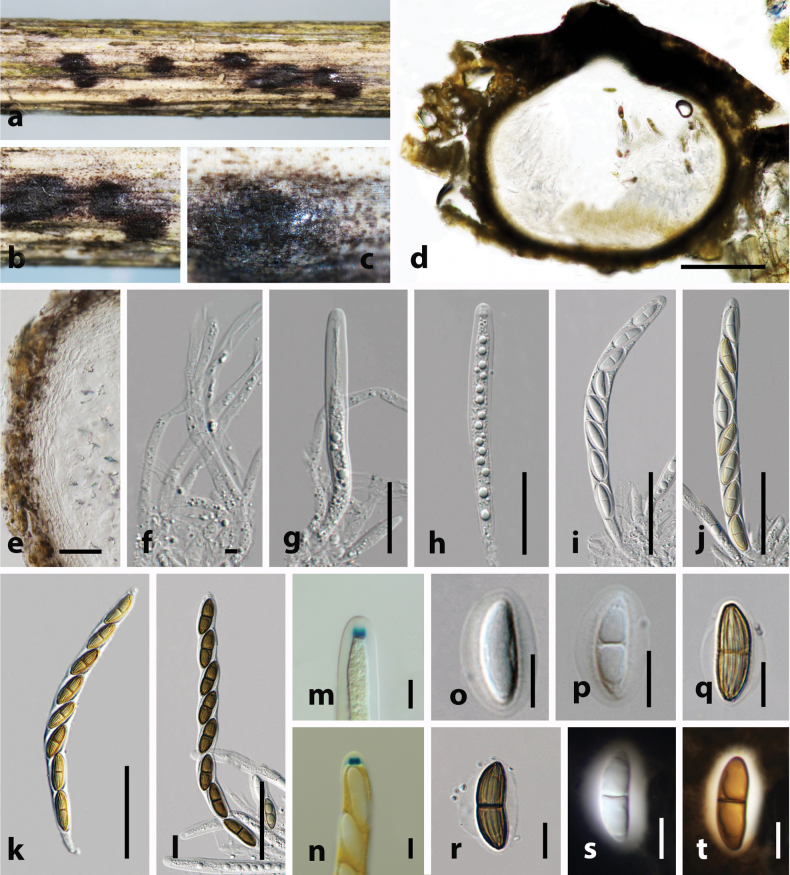
*Arecophila sichuanensis* (holotype HKAS 130468). **a**. Substrate; **b, c**. Ascomata on the host surface; **d**. Section of ascoma; **e**. Peridium; **f**. Paraphyses; **g–l**. Asci; **m, n**. Apical ring bluing in Melzer’s reagent (**m**. Immature ascus; **n**. Mature ascus); **o–t**. Ascospores; **s, t**. Immature and mature ascospores stained with Indian ink (**s**. Immature; **t**. Mature). Scale bars: 100 μm (**d, e**); 5 μm (**f, m, n**); 50 μm (**g–l**); 10 μm (**o–t**).

##### Material examined.

• China, Sichuan province, Sichuan Province, Dujiangyan City, Sichuan Longchi National Forest Park (31.075°N, 103.564167°E, 1065.01 m a.s.l.), on dead culm of Poaceae host, 5 January 2023, L. S. Dissanayake, SCCFP23-12 (HKAS 130468, **holotype**). *ibid*. SCCFP23-12 (HKAS 130469, isotype).

##### Additional GenBank accession numbers.

*btub* = PX716852, PX716853; *rpb*2 = PX733924, PX733925.

##### Note.

*Arecophila sichuanensis* (HKAS 130468) is similar to *Ar. australis* (GZUCC0124 and GZUCC0112), *Ar. clypeata* (KUNCC 23-15549, KUNCC 23-15550, GZUCC0110, GZUCC0127), *Ar. maolanensis* (GUCC 24-0116), *Ar. menglaensis* (HKAS 130461), *Ar. miscanthi* (MFLU 19-2333) and *Ar. muroiana* (GZUCC0122) having ascomata with ellipsoidal, 2-celled, guttulate, hyaline to brown, constricted at the septum, sulcate striations along the entire mature spore length, surrounded by a thin mucilaginous sheath (Wang et al. 2004; Hyde et al. 2020; Li et al. 2022; Sun et al. 2025 and this study). Phylogenetically, *Ar. sichuanensis* (HKAS 130468) clustered in a distinct clade basal to the taxa mentioned above with 71% ML and 0.97 BYPP support values. The combination of diagnostic morphology and a distinct phylogenetic placement indicates that *Ar. sichuanensis* represents an independent lineage within *Arecophila*, justifying its recognition as a separate species closely related to *Ar. australis*, *Ar. clypeata* and allied taxa.

#### Longiappendispora

Taxon classificationFungiXylarialesCainiaceae

Mapook & K.D. Hyde, Fungal Diversity 101: 139 (2020)

78573243-7416-50A3-ABFD-04537EE1B740

##### Notes.

Species of *Longiappendispora* are usually saprobic on decaying or dead wood and have been reported from tropical Asia (China and Thailand). Prior to this study, *Longiappendispora* comprised only the type species (Mapook et al. 2020). In this study, we introduce a new species, which is the second species in the genus and provides the first host record from Poaceae. It also represents the first record of *Longiappendispora* from China.

#### Longiappendispora shangrilaensis

Taxon classificationFungiXylarialesCainiaceae

L.S. Dissan. & Wanas. sp. nov.

B6B2B62F-EE35-50F9-BB11-D1CC202F51E6

861159

Fig. 6

##### Etymology.

The epithet is derived from Shangri-La, where the holotype was collected.

##### Holotype.

HKAS 130459.

##### Description.

***Saprobic*** on dead culm of bamboo host. **Sexual morph. *Ascomata*** 550–600 μm × 375–400 μm (x̄ = 577 × 390 μm, n = 5), deeply immersed under a black to dark brown clypeus, slightly raised, blackened, scattered or gregarious, globose to subglobose, erumpent, papillae in vertical section, papillae appear as black dots. ***Ostioles*** 100–110 μm × 80–90 μm (x̄ = 105 × 85 μm, n = 5), papillate, with hyaline to brown periphyses, black peridium. ***Peridium*** 40–60 μm wide (x̄ = 50 μm, n = 5), comprising several layers, outer layer brown, composed of thick-walled cells of ***textura angularis*** and inner layer hyaline, arrange with ***textura angularis***. ***Paraphyses*** 4–6 μm wide (x̄ = 5 μm, n = 10), hyaline, unbranched, cellular, aseptate. ***Asci*** 150–200 μm × 11–15 μm (x̄ = 175 × 13 μm, n = 20), 8-spored, long-cylindrical, short-pedicellate, apically rounded, with a rhomboid, J+, apical ring. ***Ascospores*** 20–25 × 6–10 μm (x̄ = 23 × 8 μm, n = 5), overlapping, uniseriate, ellipsoidal, 2-celled, guttulate (two prominent guttules on each cell), hyaline to brown, constricted at the septum, sulcate striations along the entire spore length, surrounded by a mucilaginous sheath, without appendages. **Asexual morph**. Undetermined.

**Figure 6.**
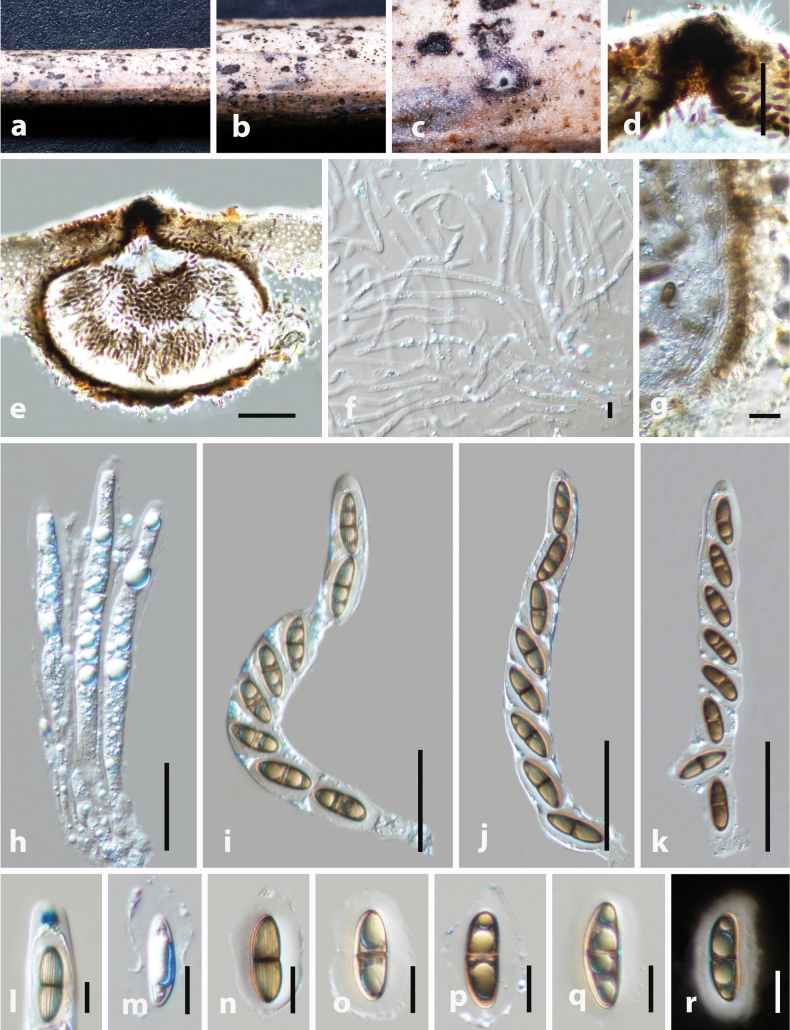
*Longiappendispora shangrilaensis* (holotype HKAS 130459). **a**. Substrate; **b, c**. Ascomata on the host surface; **d**. Section through ostiole; **e**. Section of ascoma; **f**. Paraphyses; **g**. Peridium; **h–k**. Asci; **l**. Apical ring bluing in Melzer’s reagent; **m–r**. Ascospores; **r**. Ascospore stained with Indian ink. Scale bars: 100 μm (**d, e**); 5 μm (**f, l**); 50 μm (**g–k**); 10 μm (**m–r**).

##### Material examined.

• China, Yunnan Province, Shangri-La, Deqen, skied field (27.935833°N, 99.608056°E, 2250m a.s.l.), on dead culm of Poaceae host, 18 August 2021, L.S. Dissanayake, SF18-5 (HKAS 130459, **holotype**). *ibid*. SF18-6 (HKAS 130460, isotype).

##### Note.

*Longiappendispora shangrilaensis* morphologically resembles the species of *Arecophila* having ascomata with a clypeus and 1-septate, guttulate, sulcate, and striated ascospores surrounded by a mucilaginous sheath. The phylogenetic analysis based on combined LSU and ITS sequence data indicates that *L. shangrilaensis* is closely related to *L. chromolaenae* (MFLUCC 17-1485) with 69% MBL statistical support (Fig. 1). *Longiappendispora shangrilaensis* is morphologically similar to *L. chromolaenae* (Mapook et al. 2020). But they differ in ascospores guttules and thin mucilaginous sheath (both characters absent in *L. chromolaenae*), and ascospore appendages (present in *L. chromolaenae* vs. absent in *L. shangrilaensis*). Also, the shape of ascospores of *L. chromolaenae* is different: ellipsoid to broadly fusiform, tapering towards narrow ends. Therefore, herein we introduce *L. shangrilaensis* as a novel taxon based on morpho-molecular evidence.

## Discussion

Yunnan and Sichuan Provinces in southwestern China are among the most important biodiversity hotspots in the region. Yunnan alone is estimated to harbor more than 100,000 fungal species, based on the ratio that fungal species richness is approximately six times that of vascular plants (Feng and Yang 2018). The floristic richness of Yunnan is also notable, with over half of the plant species present in China, particularly within Orchidaceae, Poaceae and Asteraceae (Qian et al. 2020). Sichuan Province, similarly, exhibits high ecological heterogeneity with mountains, basins, and varied climates contributing to its rich biota (Jiang and Zhang 2009; Xie and Yin 2022). Its southwestern region is internationally recognized for high endemism and species richness (Myers et al. 2017). Although research on fungi in Sichuan has historically been limited, recent taxonomic efforts have led to the discovery of numerous new ascomycetous taxa, indicating that the region holds significant, unexplored fungal diversity (Chen et al. 2023; Tian et al. 2022, 2023; Yang et al. 2022; Li et al. 2023; Su et al. 2023; Yu et al. 2022, 2023, 2024; Wanasinghe and Maharachchikumbura 2023; Jin et al. 2024).

Among the ecological niches that support high fungal richness, bamboo and grassland ecosystems are particularly notable. Bamboos, due to their low natural toxicity, are highly susceptible to fungal colonization and insect infestation, providing an ideal substrate for diverse microfungi (Dai et al. 2018; Wang et al. 2018; Jiang et al. 2022; Hyde et al. 2023; Zhou et al. 2024). Grasslands also serve as rich reservoirs for fungal exploration in China (Gao et al. 2024a, b), with several noteworthy species discovered on grass hosts in Yunnan, such as *Dactylella crassa*, *Hypogymnia congesta*, *Harpophora oryzae* and *Heteroconium bannaense* (Miao et al. 1999; McCune et al. 2003; Yuan et al. 2010; Xia et al. 2013; Karunarathna et al. 2017; Gao et al. 2022). Multiple species of *Anthostomella*, *Apiospora*, *Astrocystis*, *Collodiscula*, *Digitodochium* and *Microdochium* have been introduced based on collections from grasses and bamboos in Yunnan (Li et al. 2022; Dissanayake et al. 2024a; Gao et al. 2024a, b).

The present study contributes to this work by documenting novel members of Cainiaceae from bamboo and grass hosts in both Yunnan and Sichuan Provinces. The newly described species belong to genera such as *Amphibambusa*, *Arecophila* and *Longiappendispora*, and were primarily isolated from bamboo culms and grass stems, known for supporting host-specific saprobic fungi (Phookamsak et al. 2022; Yu et al. 2024; Han et al. 2024). Our findings are consistent with previous observations highlighting the substrate and host specificity of Cainiaceae and related lineages. Collections from lowland bamboo plantations in Mengla and subtropical grasslands in Shangri-La further extend the known distribution of these taxa. Some lineages demonstrated ecological flexibility, occurring in both aquatic and terrestrial microhabitats (e.g., submerged wood, decorticated culms), while others showed habitat specialization (Yu et al. 2024; Jiang et al. 2021; Zhou et al. 2024), indicating that microhabitat and host associations are important drivers of fungal diversification.

Despite these advances, the geographic scope was restricted mainly to China, which may limit the generalizability of our conclusions. Future studies should expand sampling across broader biogeographic zones to more comprehensively assess global fungal diversity. Taxonomic resolution remains constrained by overlapping morphological features and limited molecular data. While ITS and LSU are commonly used in Cainiaceae, amplification of protein-coding genes (e.g., *rpb*2, *tef*1-α, β-tubulin) is often difficult for some cainiaceous genera, which can hinder robust phylogenetic analyses. Integrative taxonomic approaches that incorporate genomics, chemical profiling, and ecological data will be crucial in resolving species boundaries and enhancing our understanding of fungal evolution and ecology.

## Supplementary Material

XML Treatment for Amphibambusa

XML Treatment for Amphibambusa hyalinospora

XML Treatment for Arecophila

XML Treatment for Arecophila clypeata

XML Treatment for Arecophila menglaensis

XML Treatment for Arecophila sichuanensis

XML Treatment for Longiappendispora

XML Treatment for Longiappendispora shangrilaensis

## Additional information

### Use of AI

No use of AI was reported.

### Author ORCIDs

Lakmali S. Dissanayake https://orcid.org/0000-0003-2933-3127

Sajeewa S.N. Maharachchikumbura https://orcid.org/0000-0001-9127-0783

Diana S. Marasinghe https://orcid.org/0000-0002-3805-5280

Ying Gao https://orcid.org/0009-0001-9830-076X

Turki KH. Faraj https://orcid.org/0000-0002-6012-8474

Jianchu Xu https://orcid.org/0000-0002-2485-2254

Dhanushka N. Wanasinghe https://orcid.org/0000-0003-1759-3933

### Data availability

All of the data that support the findings of this study are available in the main text.
